# On the Contagiousness of Entozoary Animalcules, Ascertained by Experiments, &c.

**Published:** 1846-01

**Authors:** 


Ueber die Contagiosität Eingeweidewurmer, nach Versuchen, &c.: von *Ph. Fr. Herm. Klencke*, Professor und Mitgliede der Kaiserlichen Leopoldinisch—Carolinisch Akademie, &c. &c. 8vo. pp. 168. Jena, 1844.

On the Contagiousness of Entozoary Animalcules, ascertained by Experiments, &c. By *P. F. H. Klencke*, Professor and Associate of the Imperial Academy, &c.

In the Number of this Review for April 1844, we gave a somewhat lengthened account of the researches of Dr. Klencke on a very curious and interesting subject of pathological enquiry, that of the production and propagation of Hydatidic and other entozoary animalcules in the bodies of man and of the higher Vertebrata. We there shewed that these parasitic creatures are capable of being transmitted by inoculation, in the way of direct experiment, from the body of one animal to that of another; and we adduced several other arguments to prove that the blood is the medium by which all the various kinds of Entozoa—whether Hydatidic, Intestinal, or otherwise—are usually conveyed to, and deposited in, the substance of the viscera or organs where they may happen to be found.

At the period of our notice, we had not seen this work of Klencke: our information being at that time derived from the pages of the Gazette Medicale of Paris. Having now perused the original, it has occurred to us that it may be well to recal the reader's attention to the subject, and to fill up the gaps and imperfections of our previous article. It will be useful to make a few preliminary remarks.

In Dr. Holland's excellent Notes and Reflections, there is a most interesting Chapter, headed "On the Hypothesis of Insect Life as a Cause of Disease?" Many most ingenious arguments are there adduced to shew that it is far from being improbable that certain diseases, more especially those of an epidemic and contagious nature, may be owing to the development and diffusion of minute animalcules in the atmosphere, capable, by application to the living body, of acting as a morbific virus—and thus constituting, to use the language of Klencke, a *contagium animatum*. This idea is far from being one of recent date; indeed, it was more frequently canvassed in the medical writings of last, than in those of the present, century. There are several memoirs on the subject in the Amænitates

Academicæ of Linnæus : in one, which is entitled " Exanthemata Viva," small-pox, measles, the plague, dysentery, syphilis and hooping-cough, are all attributed to animalcular infection.* And when we consider the extreme fecundity of some of the Entozoa, and consequently the excessive minuteness of their ova, there is certainly nothing at all improbable in the supposition that these germs may become introduced into the blood, and circulate through the smallest capillaries to every part of the body. Ehrenberg has hazarded the conjecture that Scrofula, in its various forms, may be thus produced—" a supposition," as Dr. Holland remarks, " not wholly new, nor incompatible with the fact of its being an hereditary disorder."

Cancer, too, has been attributed by various authors to the existence of an hydatiform body, (which, by some at least, has been believed to possess an individual and independent state of existence,) developed in those parts of the body whose vitality has become enfeebled, and whose organization therefore has began to be decomposed.

Of late years, our knowledge of the astonishing extent and prevalence of parasitic animalcules has been greatly amplified. While only eleven species of intestinal worms are recorded in the 12th Edition of the Systema Naturæ of Linnæus, nearly 1000 species are described by Rudolphi in his Entozoorum Synopsis ; and others have since been discovered. Besides this circumstance, it has been already ascertained that upwards of forty genera of insects are liable to be infested with parasitic worms (filariæ) : even within the bodies of some entozoa themselves, monades have been demonstrated to exist.—Then, again, the discovery of various

* Dr. Holland has, in the article alluded to, brought together many most ingenious arguments with the view of shewing that the origin and spreading of Pestilential Cholera were in all probability connected with the development, and dispersion through the atmosphere, of broods of invisible insects. As his work may not be in the hands of many of our readers, we are tempted to quote the following passage: " In many respects, indeed, the erratic and ambiguous course of Cholera is well represented by the flight, settlement, and propagation of the insect swarms which inflict blight upon vegetable life. Their appearance at different and often distant periods, without obvious cause for such irregularity—their direction to certain plants only—their settlement upon these in clusters and detached localities—the frequent suddenness of their change of place and final disappearance—are all circumstances of curious analogy; as also the curiously abrupt limitation of some of these swarms, shewing itself in definite lines of direction, along which their work of destruction is carried on. These lines, though to our observation seemingly arbitrary, are doubtless connected with the instincts of life under this form, and with the relation of such instincts to the surrounding media. The Cholera has been often defined in its course by similar lines of direction; sometimes stretching over considerable tracts of land or sea; more frequently obvious in particular localities; but in neither case receiving explanation from any physical conditions of the globe, from currents of air, or local circumstances of soil and climate. Nor will human communication, though certainly concerned in part in the transmission of the disorder, resolve these singularities; of which numerous examples might be cited, from the first appearance of Cholera in India, to its latest ravages in Europe. We have similar occurrences in the history of some other epidemics, but none so remarkable in degree."

sorts of animalcules in the blood of man and of the lower animals has opened a wide field for speculation. The *polystoma sanguicola* has been found in the expectorated blood of phthisical patients ; numerous *filariæ* have been repeatedly seen in the blood of dogs ; a species of *strongylus* has been observed in the blood-vessels of the horse and of the dolphin ; the *anguillula intestinalis* and *filariæ* in those of the frog ; and the *amœba* in the blood taken from the aorta of a trout.* M. Mandl has asserted that the tartar of the teeth consists of the skeletons or cases of microscopic animalcules, (Med. Chir. Rev. Jan. 1844) ; and indeed Leuwenhock had anticipated this curious announcement, as he had discovered that living *vibrios* may often be seen in the mucus of the mouth. M. Mandl suggests that the formation of the tartar may, therefore, be considered as a process altogether similar to the formation of some sandy soils, and even of certain solid slaty rocks, which have been shewn by Ehrenberg to consist entirely of the siliceous cases of animalcular insects. We need not allude to the animalcules that always exist in the seminal fluid, or to the *acarus* that is so generally present in the papules of Scabies. Our readers have no doubt heard, with a most reluctant astonishment, of late years, that almost every sebaceous follicle on the surface of the skin is the *habitat* of an animalcule ; the *acarus* or *entozoon folliculorum* of Simon and Wilson. Animalcules have been found in purulent matter ; in the mucus of the vagina, and of other passages leading outwardly ; in the gastric and intestinal juices during the process of digestion (Med. Chir. Rev. for April 1844) ; in the milk and other fluids of the body, &c.—The *trichinia spiralis* has been repeatedly discovered by many anatomists in muscular substance ; the *cysticercus cellulosa* in the same texture, and also in the substance of the nerves and brain ; a species of *filaria* in the lens and aqueous humour of the eye ; and the *distoma hepaticum* is, it is well known, a very frequent occupant of the parenchymatous substance of the liver.—We might here allude also, as being at least connected with the curious subject of *contagium vivum,* to the various instances where *fungi* and other microscopic vegetables have been discovered in the bodies of living animals. The muscardine in the silk worm, the cryptogamic fungi that have been seen in the sputa of phthisical patients, the vegetable nature of the scales in certain forms of Porrigo and Tinea, the microscopic vegetables discovered by Andral and Gavarret in albuminous fluids (Med. Chir. Rev. for July 1843), are instances of this sort ; although, certainly, it must be acknowledged that some of these alleged discoveries stand in need of confirmation. The well-ascertained fact, however, of the development of minute fungi during the processes of Fermentation and Putrefaction sufficiently attests the constant and wide-spread diffusion of germs of living matter everywhere :—all the best observers reject the doctrine of spontancous generation. Keeping these few remarks in view, the reader will, perhaps, the better appreciate the ohservations and reasonings of the author of the Contagiousness of Entozoary Animalcules : and first of Hydatids.

In our former article, we have stated that Dr. Klencke recognises five

* Vide the Medico-Chirurgical Review for July 1843 ; and for January and April, 1844.

sorts of these entozoa. These are the Hydatis Spuria—the Acephalocystis—the Echinococcus—the Polycephalus Coenurus—and the Cysticercus. It is unnecessary again to describe their peculiarities. Suffice it for our present purpose to say that, in our author's opinion, it is only the three last-named species of these cellular or cystiform productions that are to be regarded as genuine zoological objects and individual animalcules. The *spurious* Hydatid is to be viewed as nothing more than a primary cell of the organism, which has become abnormally developed, and increases itself by the generation of *Blastidia.* This cell, says Klencke, has very active endosmotic, and very feeble assimilative, powers; and thus it is that its watery contents accumulate, while scarcely any granular matter is deposited within. All the plastic activity of the increased cell, which is not employed in the process of assimilation, is expended in the production of other cellules within the original one. The latter, in course of time, bursts and discharges its brood. * * * * "I have called," he goes on to say, "these watery cell-growths 'False Hydatids,' seeing that they want that peculiar character of all true hydatids, viz: a free existence, independent of any of the organic cells of the body. The spurious hydatid, in short, is a pathological, not a zoological, object; it arises and grows just in the same manner as the cells of cancerous and other morbid growths."

We may mention also that the Acephalocystis is regarded by Klencke not as a distinct and individual Entozoon, but merely as a containing capsule or ovary of Echinococci.

There is scarcely any organ of the body in which Hydatids have not been discovered; but some are much more liable to be infested with these parasites than others: let us briefly notice them.

*Hydatids in the Brain.*

All the different species have been found in the human brain; the spurious form, however, much more frequently than the rest. As this hydatid is not a genuine parasitic animalcule, but merely an irregular primordial cellule, we are not surprised to find that often it does not give rise to any serious morbid phenomena; such as we find very generally to be the case, whensoever true Hydatids have become developed in any part of the cerebro-spinal axis. The false hydatids are rarely inclosed within a general cyst; they are usually strewed or dispersed about in groupes of different sizes. Any symptoms, which may be occasioned by their presence, seem to be the mere effects of the pressure thereby exercised upon the surrounding cerebral matter. Dr. Klencke has attempted to point out the symptoms, which may indicate the existence of the different species of hydatidic growth in the substance of the brain; but, as the attempt is more ingenious than profitable, we deem it quite unnecessary to follow his steps.

The most frequent complication of the presence of Hydatids in the Brain (and indeed everywhere,) is inflammation and its consequences. A common consequence of this is, that the hydatids become enveloped in a serous cyst. This may become so much filled with a watery effusion, as to cause very considerable pressure all around. At the same time, a good deal of fluid is usually poured out into the cavity of the ventricles.

There is one circumstance, connected with the discovery of hydatids in the brain, which deserves particular notice: it is this. Whenever Cysticerci or Acephalocysts are met with in the encephalon, we may be quite sure that we shall find the same parasites in some of the vegetative organs of the body, and in the muscles. There is good reason to suspect that the brain is affected only secondarily or consecutively. This idea is confirmed by the circumstance of the entozoa in the brain being generally less perfectly developed, and more embryonic in their appearance, than those existing in other viscera. For example, we not unfrequently find numerous Echinococci in the substance of the liver, while Acephalocysts only—the embryos, or rather the primary envelopes, of the former—are met with in the brain. Klencke alludes to one case where the liver was full of Echinococci, and a large cluster of Acephalocysts—having a number of distinctly-recognisable, half-developed, Echinococci within—was present in the left ventricle of the brain.

From the circumstance that Hydatids are very generally met with in parts which are most richly supplied with blood, and that they are usually situated close to some blood-vessels, we are naturally enough led to the conjecture that the germs of these animalcules have been conveyed to the spot, where they are found, by the circulating fluids. With respect to the *spurious* form of hydatids, which, as we have already said, are merely elementary organic cells that have become detached, and continue to retain a portion of vitality, they indeed may have been developed in the place where they happen to be found. But this cannot be the case with the true or genuine Hydatids, which are veritable animalcules and zoological individuals. They occupy, it is to be remembered, a higher position in the animal scale than many of the Infusoria; and, as they produce ova, it would be unreasonable to suppose that they are also generated in any other manner. It is by the transmissibility of these ova from one living creature to another, that we are warranted in saying that Hydatids constitute one of the elements of contagious communication.

Before quitting the subject of Encephalic Hydatids, we are tempted to quote the following case from our author.

A middle-aged man, who had been long affected with a convulsive affection of the eyelids and other parts of the face, became subject to apoplectiform attacks. The symptoms, after having continued for some time, remitted in a very remarkable manner after a free potation of brandy. This circumstance confirmed the suspicion, which our worthy author had pre-previously formed, as to the nature of the case; viz. that there were Hydatids in the brain; "as I had ascertained," says he, "by experiments, that these animalcules (Cysticerci and Polycephali) cannot endure alcohol, and either become by its use torpid, or sicken, die, and are converted into a hard, stiffened mass." (!) The patient survived for a few months—he had taken the nitrate of silver with seeming advantage—and at length died in an apoplectic attack. For some time previous to his death, he had lost the sense of smell in his right nostril, his tongue was tremulous and drawn to the left side, he stuttered in his speech, and his limbs, although not distinctly paralysed, trembled under him. *Dissection.*—In almost every part of the encephalon, numbers of Hydatids or hydatidic granulations were found. On the *falx cerebri* there was a fluctuating mass, of the size of a cacao-bean: it consisted partly of false Hydatids, and partly of Acephalocysts. The two lateral and the third ventricles were full of them. In the right *corpus striatum* and *thalamus opticus*, there were several Acephalocysts as large as peas: the cerebral matter consequently of these parts must have undergone considerable pressure. No hydatids were found in the cerebellum; but a good many Cysticerci were discovered in the *corpora olivaria* and *pyramidalia.* The *corpora quadrigemina* were so distended with water, which contained a few Acephalocysts, that all trace of the *nates* and *testes*, especially of the former, was nearly obliterated: a string of Acephalocysts extended through the mass into the left *crus cerebelli.* Acephalocysts and cysticerci were found in different parts of the lungs, and also in the muscular substance of the heart. But of all the viscera, the Liver and the Spleen, more especially the former, were those in which the hydatids—acephalocysts, echinococci, and cysticerci—were most abundant. On the outer surface of the former viscus, about its middle, but not projecting above it, there was a cyst as large as a pigeon's egg: upon opening it, a countless number of echinococci and acephalocysts flowed out. Numerous acephalocysts were observed on the peritoneal surface of the intestines, and in the folds of the mesentery. The kidneys were free; but a few cysticerci were found in the muscles of the spine.

This case is especially interesting in three points of view. 1. It shews what amount of organic deviation may be present even in the central parts of the brain, without there being occasioned nearly so much distress and suffering as might be expected: this circumstance was probably owing to the gradual and progressive supervention of the diseased formation. It is also worthy of notice, that there was no decided alteration or morbid lesion of the cerebral substance; it was nearly condensed by the presence of the numerous hydatidic bodies. 2. We observe that by far the greatest number of these bodies in the encephalon was of the nature of Acephalocysts, while the Cysticerci were most abundant in other organs, and especially in the liver. This circumstance Klencke regards as strongly confirmatory of his opinion that the liver is primarily, and the brain only secondarily, affected; in short, "that the brain is infected from the liver." 3. Although the liver and spleen contained so many hydatids, no symptom during life indicated any disturbance of the functions of these viscera.

Klencke regrets that he did not examine the blood in this case; but, at the time of its occurrence, he had not so deeply considered the subject of entozoary pathology, as he has done subsequently.

### *Hydatids of the Liver.*

All the different species of these Entozoa, with the exception of the Polycephali, has been found in this very vascular organ. As to their mode of origin, M. Cruveilhier suggests that "the Liver, which receives the entire venous blood of the abdomen, may receive along with it various imperfectly-assimilated organic molecules, which, when deposited in the cellular, or in some other, tissue of the body, may be capable of individual existence."* Such may be the case with the *spurious* form of hydatids,

* Vide the Medico-Chirurgical Review for July, 1841.

which are only detached primordial cells; but mal-assimilation cannot generate—it can merely promote the generation of—genuine hydatidic formations.

The great vascularity of the Liver at once accounts for the frequent occurrence of hydatids in its structure; for they are seldom met with in parts that are not richly supplied with blood. Their presence often gives rise to inflammation of the hepatic parenchyma, immediately surrounding them; and the usual result of this is to cause an effusion of coagulable lymph. The hydatids almost always manifest a tendency to become attached to a blood-vessel (a vein), and to have some communication with it. Occasionally a communication is established between a hydatidic cyst and some of the biliary ducts: the bile enters and kills the entozoa; or they may pass along the duct into the intestines, and then be discharged along with the fæces.

Pathologists have observed, in the case of Hydatids in the liver—especially when traces of a previous inflammation of the cyst are present—along with a deposition of a calcareous, gypseous, or cartilaginous substance, that there is often a vitreous or cheesy-looking matter that has become deposited between the different entozoa. This would seem to be a product of the inflammatory action, and to consist of fat and protein.

The so-called spurious Hydatids may at first be easily mistaken by unpractised observers for Tubercles; but their oblong, spindle shape, their granular filling up with blastidia, their transparency at first, and subsequently the existence of a cheesy matter between them—these signs will sufficiently serve to distinguish the one formation from the other.

*Hydatids of the Spleen* are usually found to co-exist with hydatids of the Liver, Lungs, and other central organs. With the exception of the Polycephali—which seem to be peculiar to the nervous substance—all the other kinds of hydatids have been found in the spleen. Indeed this very highly vascular viscus appears to be a favorite *habitat* of Acephalocysts: these creatures may there perhaps await their maturation, before they are admitted into the stream of the circulation, and become more widely disseminated. Certain it is, that this viscus often contains an immense number of very minute acephalocysts, and that they appear to be closely connected, if they do not actually communicate, with its small blood-vessels.

The *Lungs*, too, are frequently the seat of hydatids. In the *uterus*, also, they are not uncommon; generally they are situated under the mucous membrane, but occasionally in the muscular tissue, of this organ. The irritation, which they sometimes occasion in the vessels and nerves of the uterus, and the accompanying increase of size of this organ, when the accumulation of hydatids is great, may give rise to considerable ambiguity as to the cause of the symptoms in many instances of uterine disease. Cases of hydatidic formation have been, not unfrequently, mistaken for pregnancy. An instance is recorded in the Lancet for 1840, where not only the more common signs first of gestation, and then of abortion, were present, but the mammæ also became swollen, and tender, as in cases of genuine pregnancy. The development of Hydatid cysts in the *Fallopian tubes* has been known to give rise to all the symptoms of extra-uterine fœtation. They are usually associated with hydatids in the ovaria and uterus; occasionally they find their way into the cavity of the abdomen. The presence of hydatids in the Fallopian tubes will sometimes provoke the formation of a sort of deciduous membrane on the inner surface of the womb: so great is the continuous sympathy of these parts.

The *ovaria* are very frequently the seat of hydatidic formations. Sometimes the entire substance of these organs is converted into a cyst, which is full of Acephalocysts and Echinococci;—Cysticerci are rarely met with. The presence of hydatids is often associated with dropsy, hypertrophy, tubercles, &c., of the ovaria. It will, in many cases, require the aid of the microscope to distinguish with accuracy the common sacculated, from the genuine hydatidic, dropsy of the ovaria.

Klencke relates a well-marked instance of hydatids (acephalocysts and echinococci) in the *testicles*, the substance of which was wasted from atrophy. The hydatids extended upwards, along the course of the spermatic cord, into the abdomen. Small hydatidic granulations (probably false hydatids) were found even upon the nervous filaments, which proceed from the renal plexus, and accompany the cord to the testicle.

*Hydatids in the Blood.*

We not unfrequently find a sac or cyst filled with minute hydatids in the capillaries of a part: the vessel or vessels are sometimes ruptured, in consequence. This circumstance, coupled with the fact that these parasitical animalcules are only met with in those parts where there is an active capillary circulation, has led our author to the opinion that all Hydatids must be deposited from the blood into the tissue of the organ, where they may afterwards happen to be found. Cruveilhier also agrees with our author in regarding the circulation, as the medium or vehicle by which hydatids are disseminated; although (as we have already noticed) his doctrine, as to the origin of these animalcules from mal-assimilated molecules of the organism, is quite untenable. Klencke maintains that he has discovered, and been repeatedly able to demonstrate, the presence of ova in the interior of the genuine hydatids. These ova, he thinks, are originally introduced into the body from without, and infect the system by the development and dissemination of fresh ova within the body. If once a brood of hydatids find their way, either through the alimentary canal or the blood-vessels, into the tissue of any organ, there are various modes of transit by which their ova may be conveyed to other organs. In this manner alone, can we understand how various organs are simultaneously affected in almost every case, where hydatids are present in the body. Klencke alludes to those minute microscopic corpuscules which are often found in fresh-drawn blood; but he does not presume to assert that they are the ova of any animalcule. In the case of a man affected with Acephalocysts in the brain, liver, spleen, and lungs, the blood, drawn from the veins of the arm, neck, and abdomen, was found to contain not only numerous corpuscles, which in every respect resembled the ova of hydatids, but also the same small cheesy agglomerations of cells which are present in many instances of echinococci. Acephalocysts have been found in the blood of the *vena portæ;* and, as they are nothing but the ovaria of echinococci, and are often observed to be in direct communication with the adjacent capillaries, there is surely nothing unreasonable in the belief that the perfect animalcules may exist in the blood. But direct observation is not altogether wanting upon this point.

In the case of a woman, whose liver was found on dissection to contain numerous cysticerci, Klencke discovered one of these animalcules in the inferior vena cava; and an immense number of them, as well as of acephalocysts, in the blood of the splenic and portal veins, corresponding with similar entozoa in the substance of the spleen and liver. He alludes also to cases where a cluster of acephalocysts was found in one of the blood-vessels of the Pia mater;—a cysticercus in a vessel of the conjunctiva of the upper eyelid;—acephalocysts, free and loose, in the cavities of the heart;—a single acephalocyst adhering to one of the valves of the aorta;—echinococci in a varicose vein of the leg;—and lastly, where acephalocysts have been discovered in the blood which issued from a sanguineous tumour of the elbow-joint; not to mention cases where great numbers of hydatids have been found in encysted watery tumours, in various parts and organs of the body.

For these reasons, we may fairly presume, he thinks, that the blood is the medium by which the genuine hydatids are deposited in the different tissues of the living frame; unless indeed they have been inoculated or directly inserted into these tissues. Their escape and deposit from the blood may be the result either of a rupture of the capillary vessels at some point, or perhaps of suppuration being established in the part, and the consequent erosion of certain of the enveloping tissues.

Now, if we admit that genuine Hydatids are not generated in the blood, although this may be the medium by which they find their way into the various structures of the body, we must suppose that these animalcules must be introduced from without, and consequently have a *habitat* elsewhere;—in short, that they are, in strict language, as truly Ectozoa as they are Entozoa. And since they are found in animals of the most different description, it is surely not unreasonable to imagine that they may be often introduced into the human body in the animal food which is eaten. Cysticerci are especially frequent in pork; and the use of this meat may therefore be supposed to render a person liable to infection with them; more especially as I have found, says our author, that the heat of boiling water is not sufficient to destroy the vitality of their ova. It is a fact that has been clearly proved by observation, that Hydatids are often voided with the excrements; and that even the urine, the saliva, the menstrual fluid, and the milk (of cows), have been found to contain them. In short, there seems to be a continual passing of these animalcules into, and out of, the system. We are, therefore, constantly liable to become infected with them. The infection is doubtless often prevented by their being discharged from the bowels; and this usually in a dead state, in consequence of the gastric juice and bile having proved fatal to them. There is also reason to believe that certain medicines—such as Alcohol, Vinegar, Iodine, and Camphor, for example—communicate to the blood a poisonous effect upon the life of hydatids.

Some of the most active poisons seem to have little effect upon the lowest tribes of animals. Thus, the Infusoria and Polypes continue to live in a solution of Arsenic. Strychnine also and Hydrocyanic acid do not readily prove fatal to them ; and the same may be said of the alkaline sulphurets. On the other hand, the spirits of turpentine and of cubebs, creosote, and copaiba balsam are speedily destructive of hydatids. Ammonia is an active poison to them ; but perhaps the most efficient of all is Electricity, directed through the hydatidic cyst. Klencke has tried its effects not only upon lower animals, but also upon the human subject in one case, where hydatids were voided along with the urine. The use of the remedy, along with some potent diuretics, seemed to have the effect of entirely relieving his patient.

If we can believe that the ova of Hydatids ever circulate in the blood, we may understand how it should come to pass that the development of these animalcules in a part of the body often seems to be the result of a wound or injury : the ova may escape from the ruptured vessels, and gradually become developed in their accidental abode. I have known cases, says Klencke, where, in consequence of a blow upon the eye, a Cysticercus was (or at least appeared to be) formed within it; also where a large hydatid cyst—containing Acephalocysts and Echinococci—became developed in the liver, after a severe concussion of that viscus. The formation of such cysts after cutting wounds is not an uncommon occurrence. They are frequently met with in the seat of surgical operations.

Klencke maintains that hydatids may even be inherited ; their ova being transmitted from the blood of the mother to the fœtus *in utero :* in one case, he found numerous acephalocysts in the liver of a still-born child. Analogous cases have been published by Müller and other pathological writers.

What has been said above respecting the introduction of Hydatids into the body, and their multiplication within it, is, in our author's opinion, equally applicable to Intestinal Worms. All the latter entozoa are found to be provided with ova ; and every physician should know that these ova are frequently voided in immense numbers along with the mucus of the bowels. Now the question for solution is—what becomes of the ova when thus discharged ? We cannot suppose that Nature intended that they should become forthwith destroyed and annihilated. It has been shewn that the ova of intestinal worms, existing in the intestinal mucus, are not capable of being developed and matured within the bowels. They require to be first brought outward into the air, to undergo their primary stage of development, and then to re-enter the organism—not, however, by way of the bowels, but of the blood. When once in the stream of the circulation, the animalcule works its way through one of the capillary vessels into the cavity of the alimentary canal, there to undergo its last metamorphosis—that of the conversion of its entire substance into an organ of generation.* Those, who are but little acquainted with the wonderful changes and transformations which different classes of animalcules undergo, will be unwilling to

* If the view here stated be correct, we can readily understand how it should sometimes happen that intestinal worms have been found in the abdominal cavity, and even in abscesses, although no trace be discoverable of perforation in any part of the bowels. The doctrine too of the contagiousness of these parasitic animalcules is rendered, at the same time, tolerably intelligible.

admit what has been now said respecting the life of intestinal worms. But we should remember that no animal can live without having some communication (wechselthatigkeit), for a longer or shorter period of time, with the external atmosphere. This air-life, so to speak, in the case of these animalcules, appears to be that stage of their existence, which is intermediate between their existing as ova, and as living creatures, capable themselves of generating their like. When they return into the body of a living organism, they seek that part of it, which has the most direct communication with the air; viz: the blood. It is only for the purpose of depositing their ova, that the animalcules are instinctively led to take up their abode in the intestinal canal. This canal serves the same purposes to these Entozoa, that the cells of plants do to various tribes of insects which infest them.

It is well known that the excessive use of a vegetable diet, drinking of foul and unwholesome water, and residence in a moist relaxing atmosphere, more especially if this be liable to be charged with animal exhalations, and putrid effluvia, all tend to favour the development of intestinal worms. These circumstances seem to add weight to the opinion which we have expressed as to the mode of their infection. In those countries where vermination may be said to be endemic—as in Switzerland, Holland, Upper Suabia, &c.—the circumstances of external nature must tend to foster the protection and development of the animalcular ova; and the drinking of impure water afterwards, and the excessive use of vegetable food, will serve to effect their transmission to the interior of the bodies of animals. There is also good reason to believe that the prevalence of gastric typhoid fevers is apt to promote the tendency to verminal disorders.

Intestinal worms are found in all the four classes of Vertebrated animals. If we examine comparatively a great number of Amphibia, Fishes, Birds and Mammals, we shall find that these entozoa are much less numerous at certain seasons of the year than at others. At one period, they seem to disappear almost entirely from the bowels: this is usually about the end of Autumn, and in the Winter. Now, if the blood of the animal be then attentively examined, we shall often have occasion to discover in it a number of animalcules, which by some writers have been called Filariæ, by others Anguillulæ. Klencke has found these animalcules in the human blood, during the Autumn and Winter seasons; and simultaneously with attacks of alarming syncope in the individual. This relation between the disappearance of worms from the intestines, and the appearance of minute animalcules in the blood, is a circumstance that is the more worthy of notice, when we know, on the one hand, that all enthelminths undergo remarkable metamorphoses, and often change their place of residence; and, on the other, that the Filariæ or thread-like animalcules do not, in all probability, belong to one single species, but are rather the evolution-forms of different helminthal tribes. The recent researches of Miescher and Eschricht seem to prove that intestinal worms may be met with under different shapes, and in different stages of transformation.

Klencke says that he has repeatedly found in stagnant waters the same animalcules, which he has seen to exist in the blood of various Vertebrated animals: no difference in their outward features could be perceived either by himself, or by several friends who had carefully compared both species at his request. All these creatures possess the power of boring through substances of a spongy, or even of a firmer, texture. They vary a good deal in point of size; being sometimes not longer than a blood-globule, and at other times about $\frac{1}{10}$th of a line in length. Whether we may believe that these animalcules proceed from the ova of known intestinal worms, is still a problem of very difficult solution. Certainly many considerations would lead one to believe that such is the case. Their increase in the blood corresponding with their decrease in the intestines, and vice versâ, are circumstances well worthy of serious consideration.

Our author assures us that he has met with minute examples of the *trichocephalus dispar* in the stagnant water of ditches; and he therefore deems it highly probable that they, like the *lumbrici* and *tæniæ*, pass through one stage of their existence out of the living organism. We may mention also here—what we have omitted to do in a former page—that Klencke says that Hydatids are sometimes discoverable in external nature; he has found them in the water of some mineral springs, and likewise in the dung of an asparagus bed, &c. Such assertions as these however must not be received too hastily, nor without further investigations.

In the present article, we have not made any allusion to the experiments which Klencke has performed on dogs, cats, kids, frogs, &c. with the view of shewing that the various kinds of Hydatids, and also the ova of the Intestinal Entozoa, may be transmitted from one animal to another by direct inoculation. We described some of them in our previous notice of his researches; and it seems, therefore, scarcely necessary to give the details upon the present occasion. Suffice it to say that, in his opinion, the results of these experiments are conclusive as to the "contagiousness" of all parasitic animalcules.

There is one circumstance connected with the ova of Intestinal Entozoa that is more than once alluded to by our author, and may therefore deserve notice, viz: their extreme tenacity of life. The heat of boiling water does not destroy it. Even when they have been kept in alcohol for several months, or when they have perfectly dried and thus preserved for many weeks, they will revive when moistened with water. Dr. Klencke is moreover of opinion that we are not in possession of any therapeutic means that can be fairly said to be directly poisonous to, and destructive of, them; although there can be no doubt of the great utility of certain medicines in expelling the full-formed Entozoa.

The following conclusions will be found to embrace the most interesting facts (dare we call them so?), recorded in this curious production of the German press.

1. There is one species of Hydatid, represented by a semi-individual, watery Cell (*cellula primordialis hydropica sub-individuata*); it is transmissable:—we have distinguished as the "Spurious Hydatid."

2. Most of the specimens, described by practical physicians as Acephalocysts, are in truth false or spurious Hydatids.

3. Acephalocysts are not a peculiar species of Hydatids, but are the ovaria or ovi-holders of a brood of Echinococci. They arise in a threefold manner; viz: 1. The old Echinococci die off, and the congregated ova remain behind as the residuum of the old, and as the rudiment of the new generation: in such a case, we usually find that there is a common cyst or cellular envelope.—2. The Echinococci send inwardly bud-like ova, which unite with a central body, while the parent animalcules are converted into dead sacs, and form a genuine Acephalocyst: this is the usual way.—3. The Echinococci push out from their external surface gemmæ or buds, which are filled with ova: these, in course of time, become detached, and constitute Acephalocysts, capable of future generation.

4. All Hydatids are produced and propagated by *ova;* the Polycephala, however, by *budding* and *division* (gemmiparous and fissiparous); and the spurious Hydatid by *blastidia.* All of them may be communicated from one body to another, and from one organ to another; thus, their generation may take place upon a strange or abnormal bottom.

5. The pathological disturbances caused by Hydatids are dependent upon their habitat or locality, and upon their mode of life: the false or spurious sort, and Acephalocysts, never excite *periodic* phenomena.*

6. Hydatids are generally found near to a vascular plexus.

7. In the Brain, they are usually secondary: their spawn-nest (brutherd) being generally situated in some other organ.

8. They are met with free in the Blood, and are then deposited in some one organ, in consequence of the obstruction or arrest of the capillary circulation through it.

9. They are transmissible by infectious communication (ansteckung), and may therefore be regarded as a sort of *contagium animatum.*

10. The infected system has means in itself to counteract the vitality of Hydatids: there are also medicines and influences which are noxious to parasitic life, without being at all injurious to that of higher organisms.

11. The *Trichinia spiralis* is found exterior to, as well as imbedded in, the substance of the voluntary muscles.

12. It propagates itself by *ova;* thus other organisms or structures may become infected. The ova may be conveyed to various organs through the medium of the blood; their common or general cysts are often obliterated membranes of vessels, the filaments of which are discoverable even in the normal circulation.

13. The *Distoma Hepaticum* is also propagated by *ova,* and is transmissible by means of them.

---

* Klencke, in his description of Hydatids in the brain, makes this curious observation. "A diagnostic symptom that the existing Hydatids are not Acephalocysts, but are either Polycephala or Cysticerci, is the circumstance that the patient is liable to periodic attacks of pain and syncope. These appear to me to be connected with the greater or less amount of the movements of the animalcule. Every motion of its neck or of the circle of hooks around it must produce a strong irritation of the cerebral substance, and give rise to sudden accidents, which we do not observe when the Hydatids are either of the spurious kind, or Acephalocysts or Echinococci." This remark is not altogether original. An interesting case of Hydatids in the chest is recorded in the number of the Medico-Chirurgical Review for July 1841; and, in the observations appended to it, it is expressly stated that "a curious feature in this and in such-like cases is the intermittence of the symptoms, and, often too, the sudden invasion of danger, when there must have been a constant and very serious lesion of a vital organ."

14. All parasitic animals, which we have named, have a great tenacity of life.

15. *Lumbrici* and *Tæniæ,* (and by analogy) all intestinal worms, are propagated by *ova;* they must always pass one stage of their existence out of the organism, and return either as ova into the intestines of higher animals; or, having first lived through an intermediate stage as bore-worms, they find an entrance into the tissues, then seek out the blood, and ultimately undergo certain changes or transformations, according to the place of their destination.

16. Intestinal worms do not always give rise to symptoms of Vermination; the pathic or morbid condition of the bowels is alway secondary: to it we can never attribute the generation of the worms, but only the development of their already-existing germs.

17. There are various means to destroy completely-formed worms, but they have no effect upon their spawn or embryotic brood.

